# Quick algorithms for real-time discrimination of neutrons and gamma rays

**DOI:** 10.1007/s10967-014-3406-5

**Published:** 2014-08-24

**Authors:** Moslem Amiri, Václav Přenosil, František Cvachovec, Zdeněk Matěj, Filip Mravec

**Affiliations:** Faculty of Informatics, Masaryk University, Brno, Czech Republic

**Keywords:** Gamma detection, Neutron detection, Particle identification methods

## Abstract

Several new methods for the digital discrimination of neutrons and gamma-rays in a mixed radiation field are presented. The methods introduced discriminate neutrons and gamma rays successfully in the digital domain. They are mathematically simple and exploit samples during the life time of the pulse, hence appropriate for field measurements. All these methods are applied to a set of mixed neutron and photon signals from a stilbene scintillator and their discrimination qualities are compared.

## Introduction

The range of applications of neutron detectors grows fast. Nowadays, neutron detectors are used for neutron imaging techniques, nuclear research, nuclear medicine applications, and safety issues, and their usage spans on various branches of science including nuclear physics, biology, geology, and medicine. The main problem in neutron detection is the discrimination of neutrons from the background gamma rays. Fast neutrons produce recoil protons whose detection is the most common method to detect neutrons. Organic scintillators are widely used to detect these recoil protons. Fast neutrons in organic scintillators produce recoil protons through (n, p) elastic scattering and energy of a recoil proton at the highest level is equal to the energy of the neutron [1].

Among organic scintillators, stilbene and NE-213 come with some advantages for neutron spectroscopy purposes; they have rather low light output per unit energy, but this light output induced by charged protons can be easily distinguished from electrons/photons. Hence, stilbene and NE-213 scintillators produce very good results using pulse shape discrimination (PSD) methods.

Time-domain PSD methods are not computationally intensive, and hence are most suitable for real-time applications. Classically, following analog PSD techniques were most often used for $n/\gamma$-ray discrimination [2]:

1. Rise-time inspection;
2. Zero-crossing method;
3. Charge comparison.

Although analog techniques make acceptable $n/\gamma$-ray discrimination, availability of precise and fast digitizers and various PSD algorithms have made it possible to do a better discrimination of these radiations digitally. Among digital PSD methods, pulse rise-time algorithm and charge comparison are probably the most favorable ones.

In this paper, we introduce several discrimination methods and compare their separation qualities. These proposed methods are categorized into four groups: distance-based methods, area-based methods, angle-based methods, and some other simple math-based methods. To obtain the sampled data of mixed neutron and gamma-ray pulses, we use two differently-featured digitizers (explained in Sect. 2) which differ mainly in their sampling rate and output quantization level resolution. Doing so, we could find the effect of resolution and sampling frequency of the digitizers on the quality of the discrimination result for each method discussed in this article. Every experiment is carried out under the same experimental conditions, using 100,000 pulses of mixed neutron and photon signals. For this work, the field consists of mostly gamma rays and some neutrons.

A comparison among various techniques, applied to data obtained from the different digitizer types and settings, is done by using the Figure of Merit (FoM) for the $n/\gamma$-ray discrimination, defined as:

1 $$\begin{aligned} FoM = \frac{S}{FWHM_n+FWHM_{\gamma }} \end{aligned}$$

where $S$ is the separation between the peaks of the two events, $FWHM_{\gamma }$ is the full-width half-maximum (FWHM) of the spread of events classified as gamma-rays and $FWHM_n$ is the FWHM of the spread in the neutron peak [3]. FWHMs are calculated using the Gaussian fits to the neutron and gamma-ray events on experimental distribution plot.

## Experimental setup

**Fig. 1:**
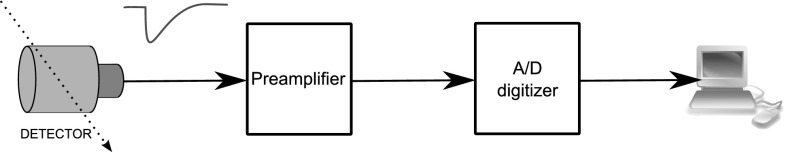
Block diagram of a digital two-parameter analyzer

The feasibility of distinguishing the detected particle types on the basis of output pulse digitization for stilbene organic scintillator, and the physics of the different time response of the neutron versus photon scintillation are known for many years. For this work, stilbene scintillation detector was used with 45 $\times$ 45 crystal, and the neutron-gamma radiation source used was 252Cf(sf). A typical scintillation detector consists of a scintillator and a photomultiplier. The latter is employed to change weak light signals impinging to photocathode (generated by the scintillator) into electric impulses. We used the photomultiplier RCA7265 [4] throughout these experiments. The block diagram of our digital apparatus is shown in Fig. 1.

A preamplifier is selected so as to match the detector output impedance. Two variants of the anode load resistance were tested in conjunction with the organic scintillation detectors. In the first variant, a load resistance of 40 $k\varOmega$ was used. A preamplifier matched it to the coaxial cable whose characteristic impedance was 50 $\varOmega$. In this case, the different waveforms of the neutron and photon pulses can be detected in the voltage pulse leading edge. If the magnitude of the load resistance is selected to be close to the characteristic impedance of the coaxial cable, which is 50  $\varOmega$, the different shapes of the neutron/photon pulses will appear to take effect during the decay time. In this case, no preamplifier is necessary. The latter option was employed here.

Two commercially available Agilent digitizers were used to digitize the output pulses: Acqiris DP210 with 8-bit resolution and set at 1 and 2 GS/s, and Acqiris DC440 with 12-bit resolution and set at 250 and 420 MS/s. While real-time digitizers are also employed in industry today, we used these specific commercial digitizers to study the effects of their various data resolution and sampling frequency features on digital processing.

## Distance-based methods

In this section, we propose several quick algorithms which are based on the distances between points on the curves of the signals and/or points on the axes of the coordination system. Such methods do not have complexity and run during the life time of the signals. One popular distance-based method is rise-time technique. In the following subsection, we review and study this method and point out the problems with it. Then, in the rest of this section, we introduce our novel methods for a higher quality discrimination.

### Classic rise-time technique

The rise-time technique, [5], [6], integrates the light pulse (e.g., of the PMT anode), and then measures the time at which this integral reaches a certain fraction of its maximum amplitude. The light output of a heavily ionizing particle, which in $n/\gamma$-ray discrimination is proton (neutron scatter interaction), has long tail; hence, the time at which this fraction is reached is longer than that of an electron (gamma ray interaction) [2]. Therefore, if the measured rise time is higher than a specific threshold, the signal is attributed to a neutron, otherwise it is attributed to a photon.

**Fig. 2:**
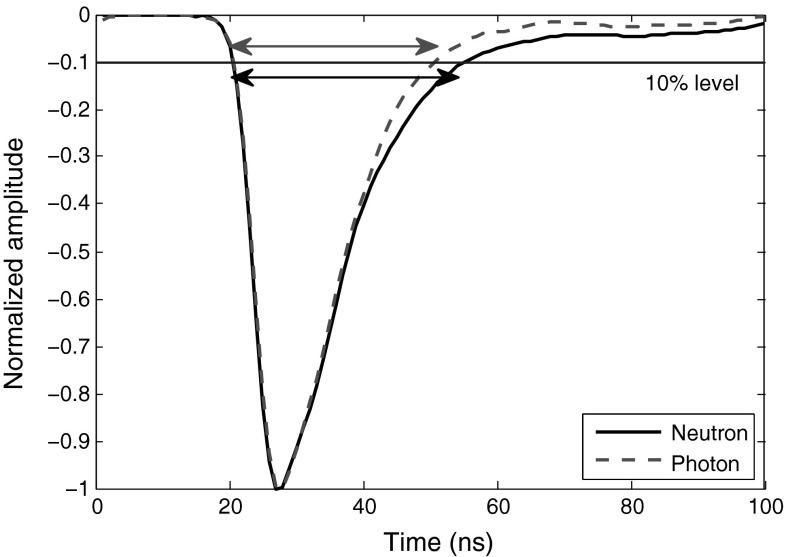
Application of “Pulse Time over Threshold” algorithm over 10 % level to sample smoothed neutron and photon signals from the stilbene scintillator

One computationally simple digital PSD algorithm is “pulse time over threshold” [7, 8], to be applied when a low anode resistor is used in conjunction with the detector. Figure 2 depicts this method applied to sample filtered neutron and photon signals obtained from the stilbene scintillator. In this case, the time during which the pulse remains over a 10 % threshold level is calculated. Depending on the noise level of the pulse baseline and the quality of the resulting signal discrimination, various threshold levels can be applied, e.g., 5 or 10%.

Since the pulses from the stilbene scintillator have fast rise and decay times, it is better to set the threshold level percentage as minimal as the maximum noise amplitude of the pulse baseline signal. This gives more room for the pulses to rise and decay, and increases the difference in measured times for neutron and photon signals. Hence, the pulses are better spread at the final plot resulting in a better discrimination.

#### DP210 digitizer (8-bit resolution, 1 GS/s)

The data obtained at 1 GHz sampling rate and recorded at 8-bit resolution contains some level of noise which should be filtered out. Our experiments show that using a 5-point moving averager removes the noise without any significant data loss. It is worth noting that data filtering is always needed, even with the lowest rate of data sampling. The higher the sampling rate is, the more level of noise reduction is required. With DP210 digitizer, while the sampling rate is high, the resolution is evidently too low to be able to discriminate the two radiation types efficiently.

**Fig. 3:**
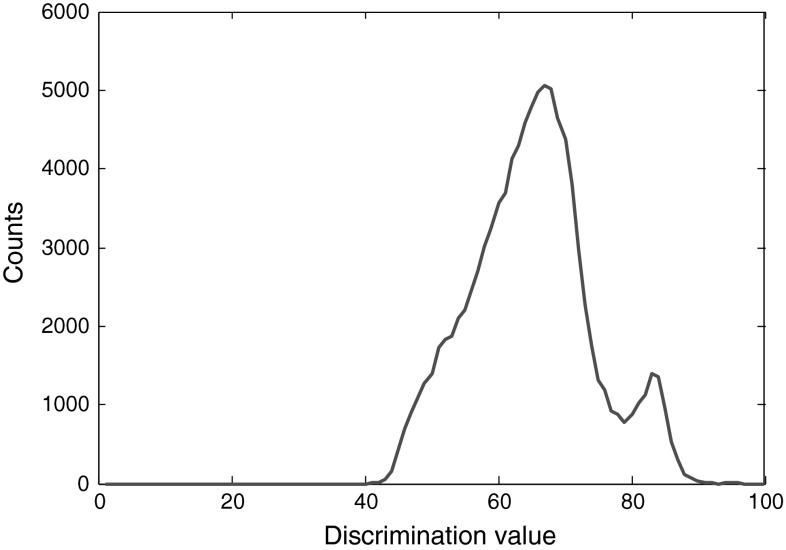
The discrimination at 2 % threshold level. The DP210 digitizer with 8-bit resolution and set at 1 GS/s is used

**Fig. 4:**
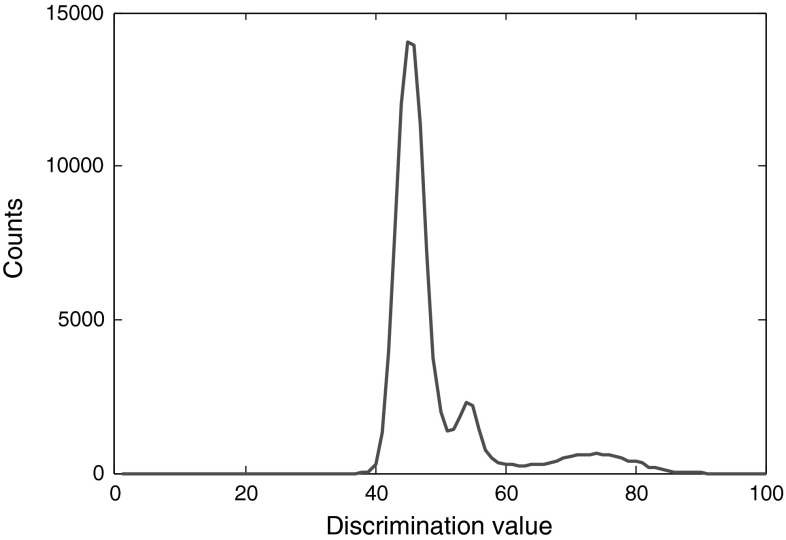
The discrimination at 4 % threshold level, illustrating the problem with “Rise-Time” method. The digitizer used is DP210, with 8-bit resolution, set at 1 GS/s

There are two problems associated with the rise-time method. First, the appropriate threshold level for discrimination varies for different data sets and the range of the suitable threshold levels for high-quality discrimination is very narrow. The best threshold level for a data set can be found through trial and error. Therefore this method is not robust. The second problem is that moving the threshold level up or down, even in small steps, could give ambiguous or non-qualified results. If the threshold level is selected too low, while the neutrons and photons fall in separate areas in the discrimination plot, each area itself could be divided into two other areas. This happens even when the data is smoothed enough. Based on our experiments, this problem exists regardless of the resolution or the sampling frequency of the digitizer. For our sample pulses, 2 % threshold level discriminates without this problem, as shown in Fig. 3, however, moving the level to 4 % gives the output shown in Fig. 4. Only through comparing with the other plots obtained at different threshold levels, do we find out that the pulses to the right of 60 discrimination value in Fig. 4 account for neutrons and the ones to the left of it account for photons. However, it is not possible to notice this discrimination by this plot alone. If we move the threshold level higher, the discrimination quality becomes too low to help us detect the two areas for the pulses. Table 1 compares the effect of some threshold-level selections for this experiment. The best discrimination takes place around 5 % threshold level. The cases where there is no proper discrimination, including the second problem mentioned above, are marked with N/A.

**Table 1:** The FoMs of “Rise-Time” method for various low threshold levels, when DP210 digitizer (with 8-bit resolution) is used at 1 GS/s

Threshold level	0.01	0.02	0.03	0.04	0.05	0.06	0.07
FoM	N/A	0.78	N/A	N/A	0.98	0.98	N/A

One solution for the problems mentioned above is to set the threshold level to higher values, typically over 10 %. However, in general, for the threshold levels higher than 10 %, the figure of merit starts decreasing, specially when the sampling frequency is low. Therefore, the quality of discrimination will not be satisfactory at higher levels. In Sect. 3.2, we propose a simple novel method to resolve this problem.

#### DP210 digitizer (8-bit resolution, 2 GS/s)

For simple calculation of rise-time technique, the number of samples between the intersection of the threshold level with leading edge and trailing edge are counted and compared for pulses. Since in this implementation, the number of samples becomes the discrimination factor, adding to the sampling rate improves the final result. However this quality improvement is not too high. Table 2 compares the discrimination quality for various levels when sampling frequency is increased to 2 GHz in DP210 digitizer. Application of a 9-point moving average filter to the sampled data has improved the discrimination quality further. The same problems mentioned in Sect. 3.1.1 also exist here.

**Table 2:** The FoMs of “Rise-Time” method for various low threshold levels, when DP210 digitizer is used at 2 GS/s

Threshold level	0.01	0.02	0.03	0.04	0.05	0.06	0.07
FoM	N/A	N/A	N/A	N/A	1.04	0.85	N/A

#### DC440 digitizer (12-bit resolution, 250 MS/s)

We repeat the rise-time method on data captured by a digitizer with 12-bit resolution and adjusted at 250 MS/s to find out the effect of the high resolution. The low sampling rate results in short-length pulses, which is 50 samples/pulse for our captured data. Despite the low number of samples, a 3-point moving average filter provides a better discrimination result than no filtering. This filtering will not cause aliasing problem at this sampling frequency. The results of discrimination proves that the high resolution brings about robustness with minimum level of noise on signal curves which compensates the weakness caused by the low sampling rate. As seen in Table 3, the range of proper threshold levels is narrow.

**Table 3:** The FoMs of “Rise-Time” method for various low threshold levels, when DC440 digitizer (with 12-bit resolution) is used at 250 MS/s

Threshold level	0.01	0.02	0.03	0.04	0.05	0.06	0.07
FoM	N/A	N/A	N/A	N/A	1.18	0.76	N/A

#### DC440 digitizer (12-bit resolution, 420 MS/s)

As our last experiment with rise-time method, we try the DC440 digitizer featuring 12-bit resolution again, but at 420 MS/s, a sampling rate higher than the one we tried in Sect. 3.1.3. Although 420 MS/s is a low sampling rate, our experiments show that 5-point moving averager works better than 3-point here. The same problems of ambiguity in low threshold level setting and low discrimination quality when this threshold is a bit higher also exist here. Table 4 compares the figures of merit for various threshold levels.

**Table 4:** The FoMs of “Rise-Time” method for various low threshold levels, when DC440 digitizer (with 12-bit resolution) is used at 420 MS/s

Threshold level	0.01	0.02	0.03	0.04	0.05	0.06	0.07
FoM	N/A	N/A	N/A	N/A	1.42	0.85	N/A

### Generalized rise-decay method

In the preceding method, the best threshold level for discrimination varies from one set of data to another and could be found by trial and error. In general, this threshold depends on the maximum magnitude of the baseline noise and also the amplitude over which the longest difference between the two pulse types exists. The best level is dependent on several factors including the physical material used, environment, and the settings of the detectors.

**Fig. 5:**
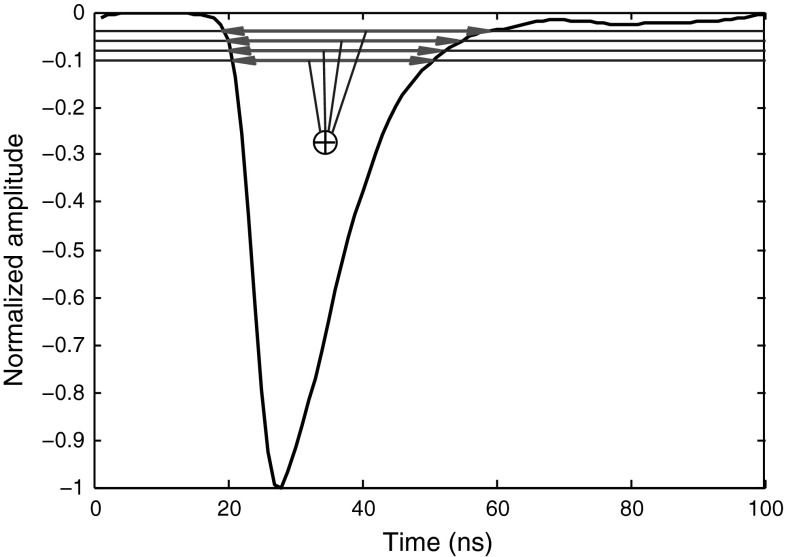
Improvement of “Pulse Time over Threshold” algorithm by adding more levels (typically, from 2 to 10 %), and summing them up. The signal shown is a sample smoothed photon

To make a general method, an alternative approach is introduced in this article which measures the time difference for a pulse at several amplitude levels and then sums them up. While this approach keeps the calculation simple, it overcomes the problems already mentioned in Sect. 3.1.1. Studying various pulses proves that in general the largest difference between the two pulse types is within the low 10 % level range. As we move the threshold level further up toward the peak, the difference becomes smaller. Around 50 % threshold level, this difference is almost negligible (for the data obtained at higher sampling rate, the difference is a bit larger than the ones obtained at lower sampling rate). Including the time difference within the higher amplitudes usually will not improve the final discrimination. In fact, because from around 50 % level to the peak, the two radiation type signals are almost the same, including this upper half of the signal equals addition of a constant value to the discrimination factor, which will decrease the overall discrimination improvement. The best result is obtained when we appoint levels within the range of 2–10%; we appointed 9 levels in this range. Figure 5 illustrates the application of this method to a sample smoothed photon pulse. Table 5 compares FoMs for the data obtained through different digitizers, and each at different settings, when this method is applied.

**Table 5:** The FoMs obtained when using “Generalized Rise-Decay” method on the data captured by digitizers with different resolutions and sampling rates

Digitizer	8-bit, 1 GS	8-bit, 2 GS	12-bit, 250 MS	12-bit, 420 MS
FoM	1.26	1.20	1.16	1.24

### Basic amplitude difference

**Fig. 6:**
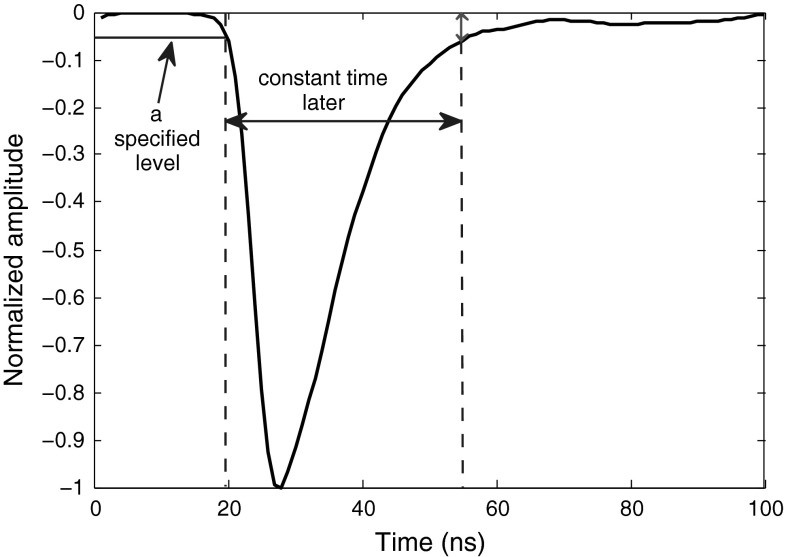
Application of “Basic Amplitude Difference” method to a sample smoothed photon pulse

This method, introduced in this paper, takes advantage of the difference in energy levels of neutron and gamma pulses at a certain fixed point in time within the trailing edge of the pulse. To implement this method, a specific starting point should be assigned for every pulse, and some constant time after this starting point should be marked and the levels of the pulses at the marked points compared. While the peak of a pulse seems to be the best starting point, irregularities on the peaks of the pulses make it difficult to use it as a solid starting point. The best choice for starting point would be a specific level within leading edge. Since there is almost no difference between the leading edges of the neutron and photon signals, selection of any level as the starting point on the leading edge of the signals would provide the same result. The constant time after this starting point should be set such that it falls within low 10 % fraction of the amplitude on the trailing edge of the pulse which makes the highest possible energy difference between the two pulse types. In our experiments, we use some training pulses to find the best interval. First, the intersection of a fixed threshold level (e.g., 20 % level) and the leading edge of every training pulse is used as the starting point. Then, the intersection of the trailing edges of the training pulses and a 5 % level are found and used as the ending points. The distance between the starting point and the ending point for every training pulse is found, and these distances are averaged, resulting in a constant value. For all the pulses in the experiment, we find the starting point, in the same manner we found it for the training pulses, and then this constant value is added to the starting point, giving the point whose energy level should be used as the discrimination factor. Since this method compares a small fraction of the pulse peak-amplitude for neutrons and photons, the range of normalization of the pulse signals should be large enough so that this fraction is scaled to a large value, and hence could be used as a proper discrimination factor. Figure 6 illustrates the application of this method to a sample filtered photon pulse. Table 6 compares the FoMs of four data sets captured under different settings using this method.

**Table 6:** The FoMs of “Basic Amplitude Difference” method for digitizers with different resolutions and sampling rates

Digitizer	8-bit, 1 GS	8-bit, 2 GS	12-bit, 250 MS	12-bit, 420 MS
FoM	0.83	0.85	0.95	0.99

Unlike the rise-time method in which moving the threshold level even in small steps could result in ambiguous or non-qualified discrimination, here in this method, the time range within which the curve values can be compared and still give acceptable results is wider. However, the FoMs obtained are less than the ones in rise-time method, as Table 6 shows. In the following section, we try to improve this simple method.

### Generalized amplitude difference method

**Fig. 7:**
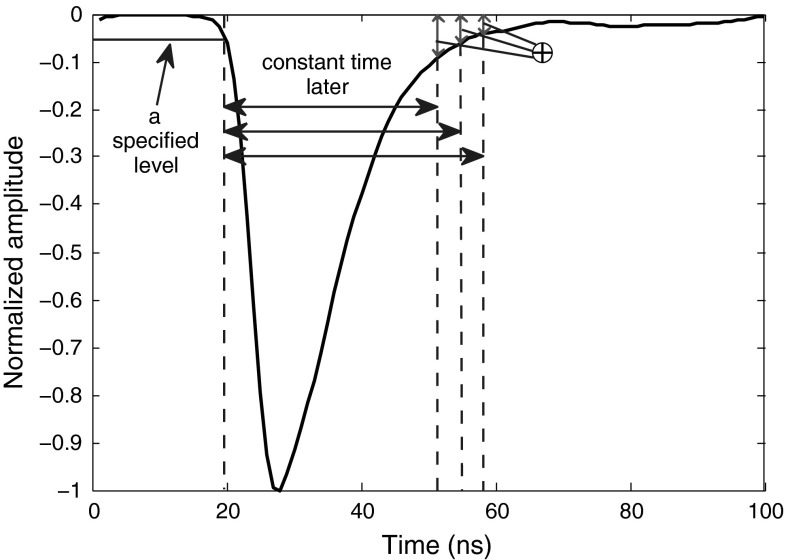
Adding amplitudes of a pulse at several points in time. The starting point of the intervals is when the signal reaches a specific level on the leading edge. The *pulse* shown is a smoothed photon

In the preceding method, the intersection points of a specific threshold level and the back edges of some training pulses are used to help find the time at which a pulse amplitude is to be measured. The specific threshold level selected could be ideal for some datasets (obtained under specific experimental settings), but not necessarily for all. Therefore, instead of exploiting only one point within the back edge, the best general approach which also keeps the calculation simple for field measurements, is to capture the amplitudes at several points in time (within the low 10 % energy level of back edge) and add them up. In our measurements, we use some training pulses to find several averaged time intervals which begin from a fixed point at front edge. Every averaged interval is found using the approach explained in Sect. 3.3. In our implementation of this method, we measured nine averaged values in total, corresponding to the levels from 2 to 10% (in steps of 1 %) which intersect with the back edge of every training pulse. Then, for every pulse in the experiment, starting from the specified level on the front edge, the signal’s amplitudes after these averaged values are obtained and summed up, and the resulting value is treated as the discrimination factor. Like the previous method, the signals should be such normalized that the discrimination factor is not too small. Figure 7 shows the application of this method to a sample filtered photon pulse. The FoMs obtained using this method are satisfactory, as shown in Table 7. The robustness of this method on various datasets is its main advantage.

**Table 7:** The FoMs of “Generalized Amplitude Difference” method for digitizers with different resolutions and sampling rates

Digitizer	8-bit, 1 GS	8-bit, 2 GS	12-bit, 250 MS	12-bit, 420 MS
FoM	0.99	0.93	0.94	1.09

### Two dimensional method

**Fig. 8:**
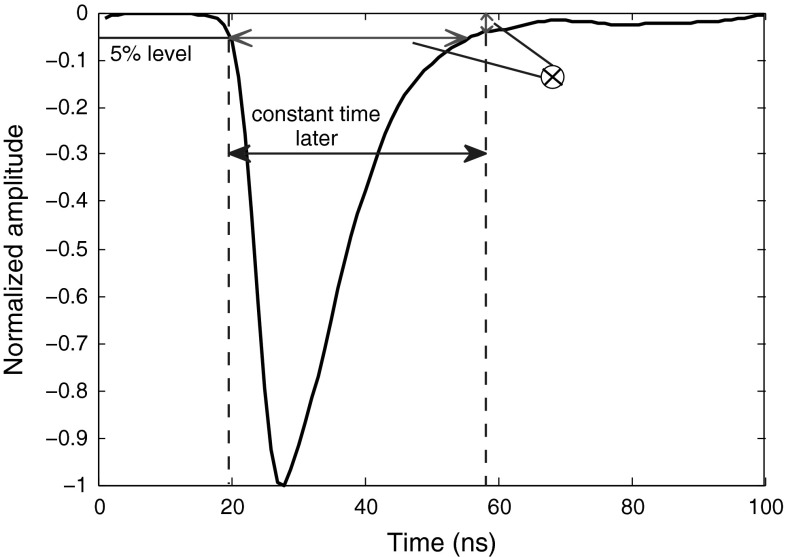
Application of “Two Dimensional” method to a sample smoothed photon signal

Every method discussed so far takes advantage of difference between the shapes of neutron and photon signals only in one direction; either horizontally through the difference in their timing, or vertically through the difference in their energy levels at fixed points in time in their life. Obviously, a better approach is to exploit both these differences. A simple, yet efficient, method is to combine this time value with the energy level value of a signal and use it as the discrimination factor. Although addition would work, multiplication better reflects the difference between these two radiation types. Figure 8 illustrates this method where a value obtained through rise-time method at 5 % level is multiplied by the energy of the signal at a predefined timing point.

As Table 8 shows, this method provides a high quality discrimination. Besides, this method gives a highly accurate results due to considering the differences between radiation types in two dimensions. A study of the neutron and photon pulses shows that there are cases where a pulse type is different than the other type only in one of the time or energy coordinates, not both. This method provides a more accurate discrimination result in such cases.

**Table 8:** The FoMs of “Two Dimensional” method for digitizers with different resolutions and sampling rates

Digitizer	8-bit, 1 GS	8-bit, 2 GS	12-bit, 250 MS	12-bit, 420 MS
FoM	1.01	1.03	0.94	1.10

### Distance on trailing edge

Exploiting the curve of a pulse in both coordinates of time and amplitude provides an efficient result. In preceding section, we used such a method for discrimination. An ideal approach which captures a large difference between the two radiation types is to cross a straight line, with a positive slope, to the trailing edge of the pulse curve, such that the two intersections occur within its low energy segment. The distance between the two crossing points would be an efficient discrimination factor. However, finding this line with positive slope should be done with trial and error, and once a fixed line is found, there is always the possibility of facing a pulse which does not cross the line at any point. Therefore, this is not considered a solid method.

**Fig. 9:**
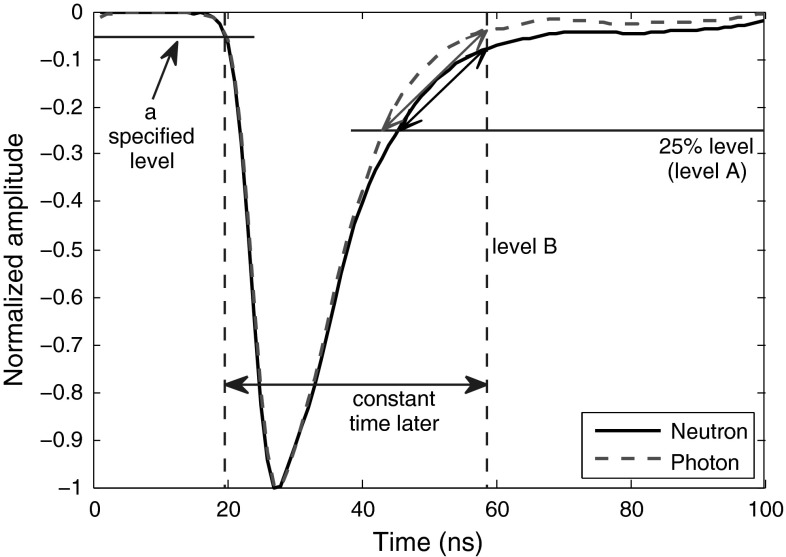
Application of “Distance on Trailing Edge” method to sample smoothed neutron and photon signals

We propose a reliable method in this article in which the distance between two points on the curve is the discrimination factor (illustrated in Fig. 9.) One point is the crossing of the curve and a perpendicular line to the x-axis (shown as level B in Fig. 9) which has some constant distance from a specified level on the leading edge. For our captured data, this crossing point on the trailing edge makes acceptable vertical difference between neutrons and photons when it falls at about 5 % level amplitude of the pulse. The constant value (to be added to the starting point) can be easily obtained; working on some training pulses, we specify a fixed starting point on the leading edges of these pulses, then the crossings of trailing edges of these pulses and a 5 % threshold level are detected (called ending points), and finally the intervals between the starting points and ending points of these training data are averaged, giving the final value. The second point is found by using a perpendicular line to the y-axis (shown as level A in Fig. 9) which crosses the curve at a point where it leaves some difference between the two radiation types. The difference between the two radiation types at the crossing point of the curves with level A is crucial. Our experiments show that for the data obtained at lower sampling rates, this difference is not enough to result in an acceptable FoM, even when level A is moved to lower levels. Therefore, this method directly depends on the sampling rate of the pulses. For the higher sampling rates of 1 and 2 GS/s, we set level A at 25 %; the FoMs obtained are shown in Table 9. For the low sampling rate of 250 MS/s, this method fails to provide a proper discrimination, and for 420 MS/s sampling rate, the best result is obtained when we move level A line lower to 15 %, resulting in FoM of 0.84, which is not efficient.

**Table 9:** The FoMs of “Distance on Trailing Edge” method for digitizers with different resolutions and sampling rates

Digitizer	8-bit, 1 GS	8-bit, 2 GS	12-bit, 250 MS	12-bit, 420 MS
FoM	1.18	1.73	N/A	N/A

**Fig. 10:**
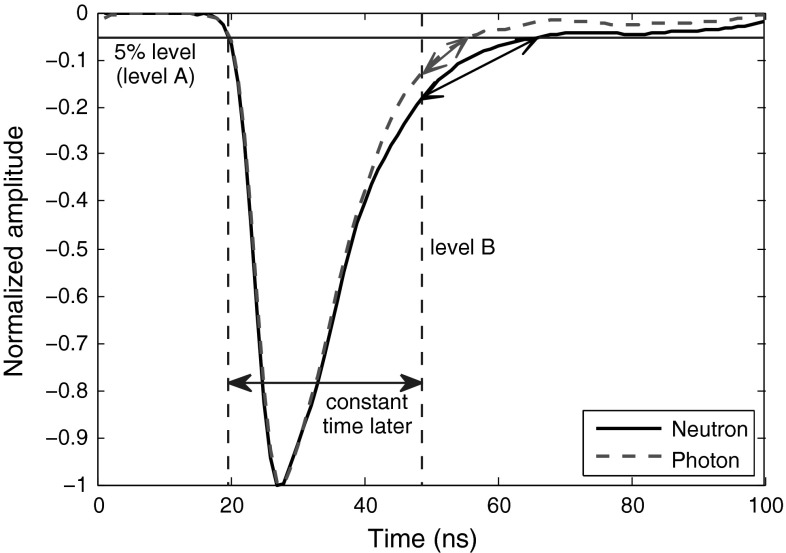
Application of revised “Distance on Trailing Edge” method to sample smoothed neutron and photon signals

The method introduced above is too sensitive to the sampling-frequency. To bring balance to the method, we can alternatively use the approach explained in Fig. 10. Table 10 shows FoMs of this modified version. The FoMs in Table 10 are all found when line A in Fig. 10 is set to cross the signals at 5 % level, and line B crosses the signals at a constant point in time within their rise times, where this crossing is trained to occur approximately at 15 % fraction of their amplitudes, averagely. However, the overall rule is that the higher the sampling frequency is, the closer the line B should get to the peak to provide better discrimination. For example, for 250 MS/s, line B at 10 % provides some discrimination, for 420 MS/s, line B at 12 % works better, for 1 GS/s, line B at 15 %, and for the 2GS/s, line B at 30 % level is better. However, since in this version of the method, the crossing of level A with the curve (which contributes more to the final FoM result) is at 5 % level and therefore less dependent on sampling frequency, the movement of level B (which is less important in the final FoM calculation, but directly dependent on the sampling frequency) does not affect the result much, unless the sampling frequency is too low as in the case of 250 MS/s in Table 10. This feature makes this approach solid and more general.

**Table 10:** The FoMs of revised “Distance on Trailing Edge” method for digitizers with different resolutions and sampling rates

Digitizer	8-bit, 1 GS	8-bit, 2 GS	12-bit, 250 MS	12-bit, 420 MS
FoM	1.18	1.07	N/A	1.13

## Area-based methods

### Numerical integration within trailing edge

**Fig. 11:**
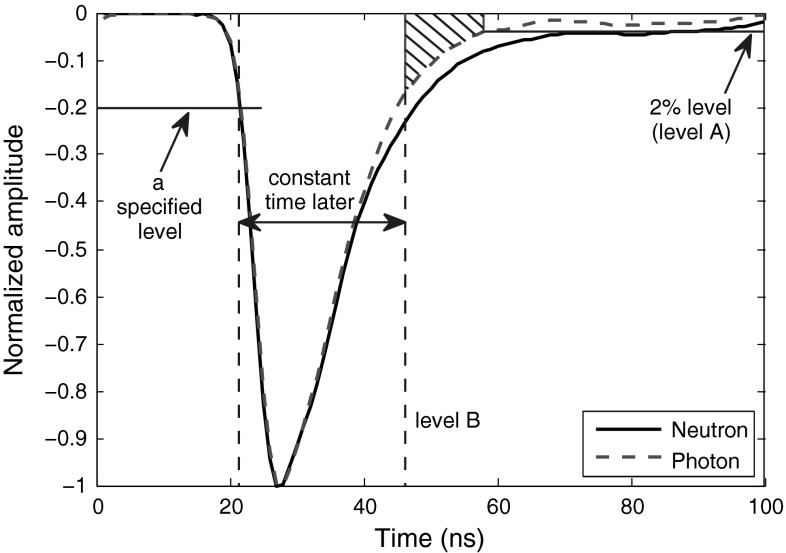
Application of “Integration” method to a smoothed photon pulse

Another efficient method is to discriminate the two radiation types by comparing the numerical integration of a section of the trailing edge of their curves. The application of this method to a sample filtered photon pulse is shown in Fig. 11. There are two levels cutting the curve of a pulse and hence marking the beginning and the end of the section whose integral is to be measured. Level A is perpendicular to y-axis in order to make the area under photon pulse smaller than the area under the neutron, and hence make the integration difference between these two pulse types larger. When applied to our data, level A set at 2 % provides better results. Level B, which marks the other end of the curve cut, is perpendicular to the x-axis so that the energy difference between the two radiation types could make its effect on the discrimination factor. This level occurs a constant time after a specified level on the leading edge of the curve; this distance in time is obtained for some training pulses (starting and ending at specified threshold levels on the leading and the trailing edge), and then these values are averaged, and the result is used as a constant value for the pulses of the whole data set.

Table 11 shows the FoMs for various level Bs (i.e., the threshold level set on trailing edge, when calculating level B), when this method is applied to the pulses obtained using the digitizer with 8-bit resolution and 1 GS/s frequency rate. As seen in this Table, the FoM when level B is close to the peak is low. The reason is that the curves of neutrons and photons fall on each other for almost the top half fraction of the amplitude (this is seen in Fig. 11). This similarity between the curves of neutrons and photons within the top part of their trailing edges extends more through the curve for the pulses obtained at lower sampling frequencies. Including this common part between neutrons and photons, which has a large integral value, results in a large constant value to be added to some varying value which corresponds to the area of the low 50 %, and hence reduces the final discrimination efficiency. As we move level B down to 50 %, this constant value gets smaller and therefore FoM gets better. From almost 50 % level B down, the energy difference between the crossing points of level B and the curves of neutron and photon pulses gets larger, resulting in better FoM. As seen in Table 11, the best result is obtained when level B is set at about 20–40 % for the pulses obtained with DP210 and at 1 GS/s. Table 12 shows the FoMs for the other data sets when level A and level B (as the two limits for integration) are set at 2 and 20%, respectively.

**Table 11:** FoMs of “Integration” method for the pulses obtained from the digitizer DP210 (8-bit, 1 GS/s), when the area is bounded by 2 % level A (in Fig. 11) and by various level B percentages as shown in the Table

	Peak	90 %	80 %	70 %	60 %	50 %	40 %	30 %	20 %	10 %
FoM	0.94	1.00	1.05	1.08	1.13	1.11	1.20	1.21	1.23	1.15

**Table 12:** FoMs of “Integration” method for the pulses obtained from various digitizers, when the area is bounded by 2 % level A (in Fig. 11) and by 20 % level B

Digitizer	8-bit, 2 GS	12-bit, 250 MS	12-bit, 420 MS
FoM	1.21	1.10	1.22

## Angle-based methods

Another efficient approach to discriminating neutron and photon pulses is use of angles in the measurements. Angle-based methods prove to be more sensitive to the differences between pulse types. The vertex of an angle can easily be placed at the best point on the curve coordinate system to provide us with high quality discrimination. Discrimination quality of angle-based methods are easily affected by the curve smoothing approach, hence, filtering of the signals should be done properly.

### Based on time difference (horizontal difference)

**Fig. 12:**
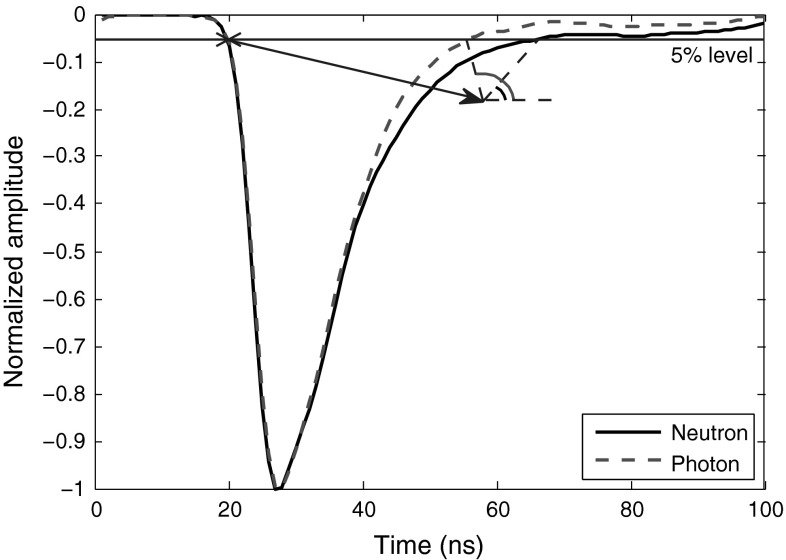
Application of “Angle-Based” method (horizontal difference) to smoothed neutron and photon pulses

An efficient discrimination of neutron and photon pulses can be achieved by measuring an angle whose vertex is close to the crossing point of the pulse curve trailing edge and a perpendicular line to the y-axis. One arm of this angle could be perpendicular to the y-axis and the other arm pointing to the said crossing point. Figure 12 shows this implementation. In this implementation, a 5 % level is crossed with the leading edge of the curve. Then this crossed point is shifted forward a constant amount (on x-axis), and downward a constant amount (on y-axis). The constant movement on x-axis is such that it falls between the range of two points obtained by crossing a 5 % level with the trailing edges of a neutron and a photon signal. To do so, some training signals are used, similar to the cases we had in the preceding methods. The constant movement on y-axis must be such that the vertex is not either very close to the crossing of 5 % level and the curve (because the final discrimination plot would be so scattered), or very far from the crossing (which will result in low-quality discrimination). Y-axis movement of the vertex is also dependent on the sampling frequency, e.g., for discrimination of data obtained at 1 and 2 GS/s, we moved the point to $-60$ and $-120$, respectively. Table 13 shows the FoMs when applying this method to the data sets obtained from various digitizers.

**Table 13:** FoMs of “Angle-Based” method (horizontal difference) for the pulses obtained from various digitizers

Digitizer	8-bit, 1 GS	8-bit, 2 GS	12-bit, 250 MS	12-bit, 420 MS
FoM	1.16	1.11	1.25	1.39

### Based on energy difference (vertical difference)

**Fig. 13:**
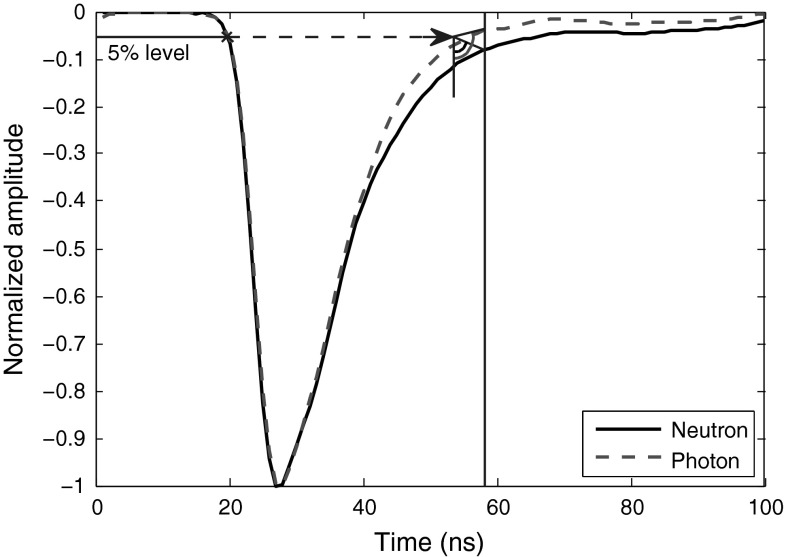
Application of “Angle-Based” method (vertical difference) to smoothed neutron and photon pulses

The preceding angle-based technique can also be employed to discriminate based on the difference between the energy levels of the two pulse types at some fixed point within their rise times. To implement this method, as shown in Fig. 13, the crossing of the 5 % level and the leading edge of the curve is marked and then shifted forward a constant amount to be the vertex of an angle with one arm pointing to the crossing of a perpendicular line to the x-axis (occurring a constant time after a specified level on the leading edge) and the curve, and the other arm directing downward perpendicular to the x-axis. The y-axis of the curve should be scaled properly to provide us with quality discrimination. Because this method works on the energy level of the pulses, the discrimination is much better for the data obtained using a digitizer with a high resolution, as Table 14 proves this.

**Table 14:** FoMs of “Angle-Based” method (vertical difference) for the pulses obtained from various digitizers

Digitizer	8-bit, 1 GS	8-bit, 2 GS	12-bit, 250 MS	12-bit, 420 MS
FoM	0.88	0.92	1.06	1.34

## Other simple methods

### “Mean vs. standard deviation” method

**Fig. 14:**
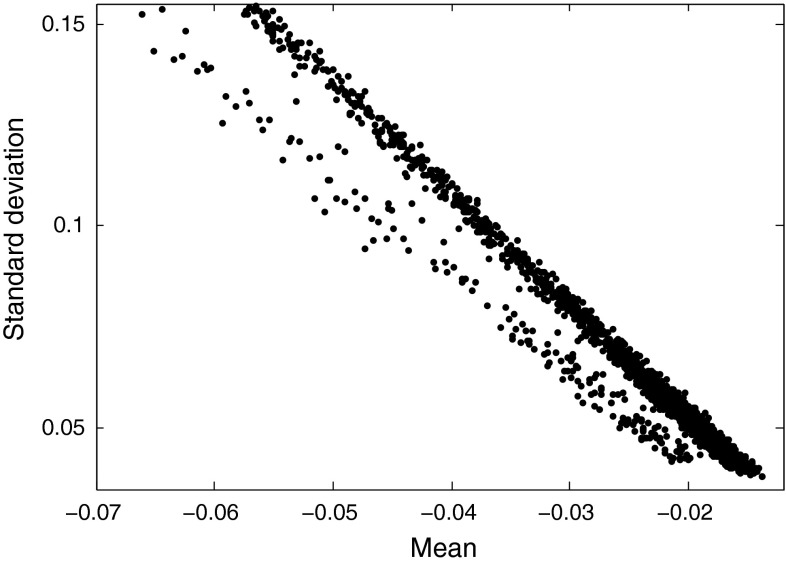
Plot of “Mean vs. Std.” of the mixed pulses obtained using the digitizer with 12-bit resolution and 420 MS/s

Analyzing the features of neutron and photon signals reveals that the plot of mean vs. standard deviation (std.), or mean vs. variance (var.), of the mixed pulses could be used to provide an excellent discrimination factor. While both of these methods provide decent discrimination results, the pulses of the two radiation types on the mean vs. var. plot are lined up in a curved fashion, while on mean vs. std. plot, they are grouped in two straight lines, as shown in Fig. 14. Therefore, our focus in this section is on the latter one.

Another property of this method is that it reveals the pulses which have not been recorded properly. If the number of register bits are not enough to cover the whole amplitude range of the high-energy pulses, overflowing will happen while sampling the region around the peak of the pulse. For such pulses, the mean vs. std. relation will be different than the relation for correctly recorded pulses. These pulses do not follow the straight lining pattern on the mean vs. std. plot, and hence can be easily distinguished.

“Mean vs. Std.” method has the following advantages:

1. Normalization of the pulses is not required;
2. No noise filtering is necessary, since mean and std. both contain average filtering properties;
3. Mean and std. can be processed quickly using running statistics while receiving every new sample from the digitizer, without requiring all the samples to be involved in each new calculation. This feature makes mean vs. std. method ideal for real-time processing.

The mean of a signal contained in $x_0,x_1,\ldots ,x_{N-1}$ is calculated as:

2 $$\begin{aligned} mean = \frac{1}{N}\sum _{i=0}^{N-1}x_i \end{aligned}$$

and the std. as:

3 $$\begin{aligned} std&= \sqrt{\frac{1}{N-1}\sum _{i=0}^{N-1}(x_i-mean)^2}\nonumber \\&=\sqrt{\frac{1}{N-1}\left[ \sum _{i=0}^{N-1}x_i^2-\frac{1}{N}\left( \sum _{i=0}^{N-1}x_i\right) ^2\right] } \end{aligned}$$

Since the number of samples for every signal is constant in an experiment of neutron and photon discrimination, on receiving every new sample of a signal, only two parameters need to be updated: the sum of the samples received so far, and the sum of the square of the samples received so far. Upon receiving the last digitized sample of the signal, the values of these two parameters are used for the calculation of mean and std. based on the Eqs. 2 and 3.

Table 15 shows the FoMs of “Mean vs. Std.” method for the data obtained via various digitizers. As seen, the results are solid; this method discriminates well irrespective of the digitizer features.

**Table 15:** FoMs of “Mean vs. Std.” method for the pulses obtained from various digitizers

Digitizer	8-bit, 1 GS	8-bit, 2 GS	12-bit, 250 MS	12-bit, 420 MS
FoM	1.13	1.10	1.15	1.11

### Application of FFT method

**Fig. 15:**
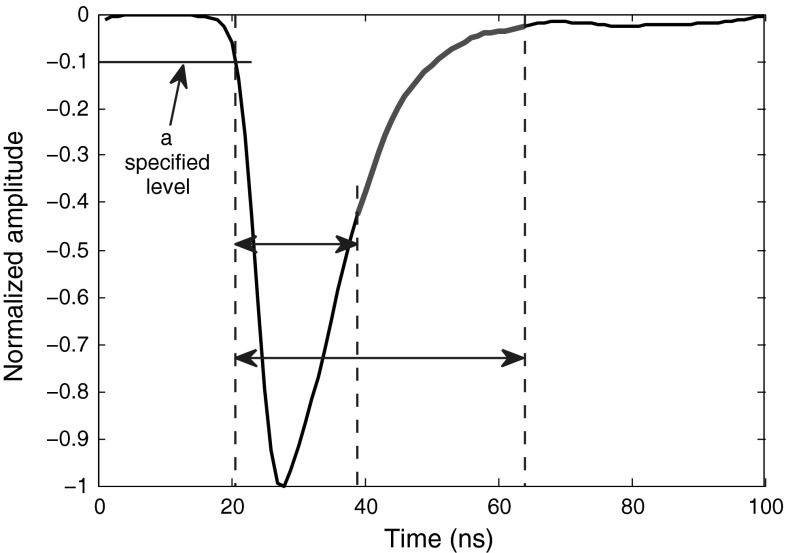
Segment of an unknown signal, shown in *bold red*, to be used in our discrimination method

The trailing edge of the neutron signal has higher rise time than that of the photon signal. However, this difference is not large enough to be exploited by directly applying signal processing techniques. In this Section, we introduce a discrimination method based on the frequency-domain data. We apply FFT (fast Fourier transform) only to a short segment of a normalized unknown pulse which is different between neutrons and photons (Fig.  15); the two ends of this segment are determined by adding two constant amounts of time to the point when a specified level on the leading edge is reached. Determination of a specified level on the leading edge as the starting point is arbitrary because the two leading edges of neutrons and photons are almost the same. Some training pulses are used to find the two constant amounts of time after the starting point within which the differing segments of neutrons and photons exist. In the experiments we carried out, this segment fell on the trailing edge from about 2–40 % of peak-amplitude, on average. However, changing these boundaries will not have a significant effect on the result. Given this segment, the following steps are taken:

1. Hamming window is applied to the said segment of the normalized pulse;
2. Mean of the windowed curve is subtracted from every point;
3. The signal is padded with enough number of zeros to make the total number of points a power of two;
4. FFT is taken.

In Step 1, the Hamming window is used because it is raised on a pedestal. This property of Hamming window helps retain the sloped shape of the cuts of the two radiation types (red segment in Fig.  15) as much as possible. As we will explain, this sloped shape helps exploit the differences between the radiation types.

**Fig. 16:**
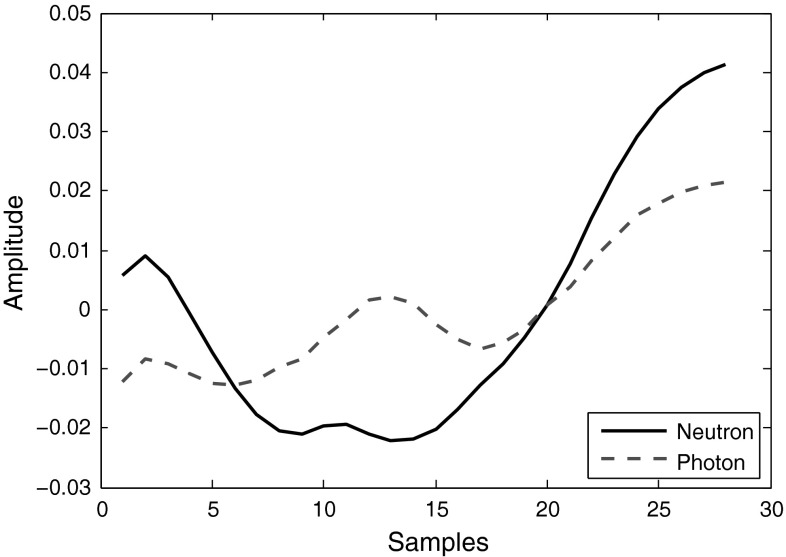
Neutron and photon signal segments, obtained under 12-bit resolution and 420 MS/s sampling rate, after mean of the windowed curve is subtracted from every point

**Fig. 17:**
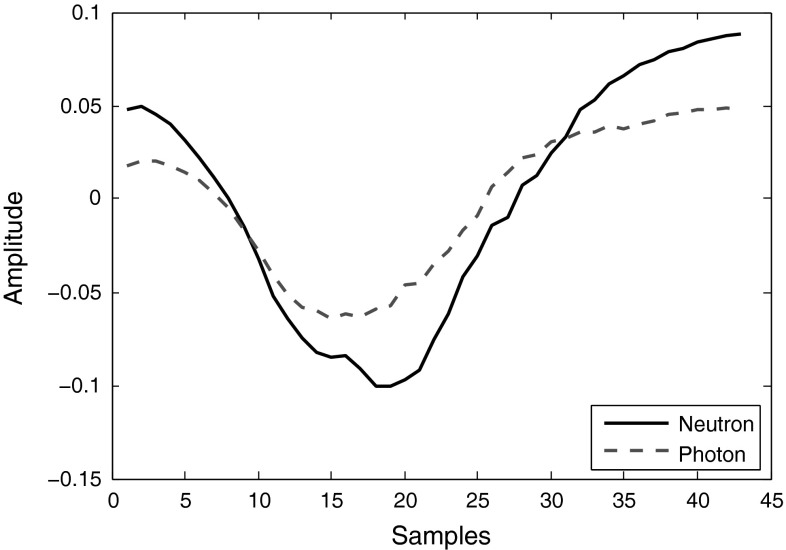
Neutron and photon signal segments, obtained under 8-bit resolution and 1 GS/s sampling rate, after mean of the windowed curve is subtracted from every point

In Step 2, the mean of curve is subtracted from every point *after* application of window, while typically this is performed *before* window application (for removal of DC spectral component). This will cause the left ends of the neutron and photon pulses get opposing amplitude signs, as Fig.  16 illustrates. Since the samples with lower indexes have higher frequencies, the different signs of neutrons and photons will create a mirror image of them in high frequency region of their spectra. Although this difference could be easily used for discrimination, mid-samples in Fig. 16, which contribute to the lower frequencies, have this property too. This difference is not always achievable: it depends on the resolution of the data, the length of the segment used, and the instrumentation settings. Figure 17 shows the result of the same approach applied to the data obtained with 8-bit resolution digitizer set at 1 GS/s sampling rate. Digital signal processing techniques almost fail to discriminate in such cases. In Sect.  6.3, we will introduce a general approach to resolve this issue.

**Fig. 18:**
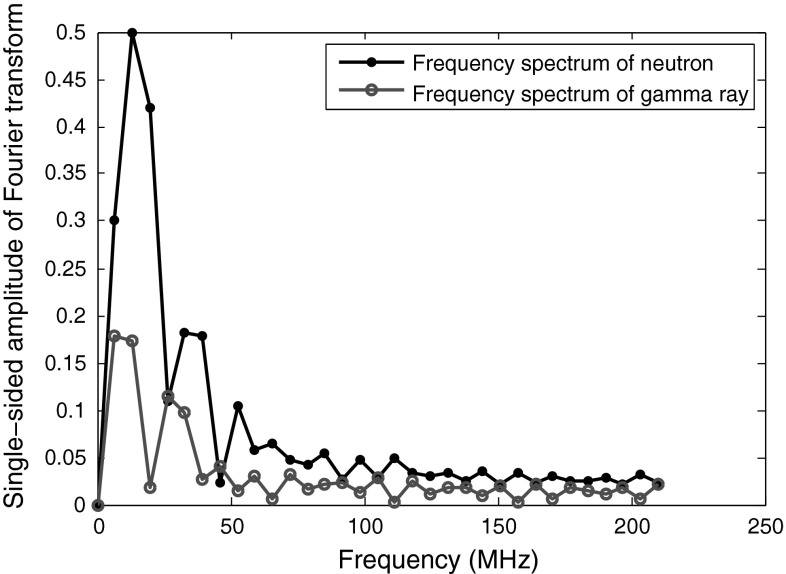
The magnitude spectra of $\gamma$-ray events and neutron events, applying a 64-point FFT. The pulses are obtained using DC440 digitizer (12-bit resolution, 420 MS/s)

Figure 18 shows the magnitude spectra of two sample neutron and photon pulses obtained by DC440 digitizer (set at 420 MS/s). As seen in this Figure, the peaks of the lobes of the $\gamma$-ray pulses have lower frequencies than neutrons (specially in low-frequency region). This fact could be easily used to distinguish the two signals. However, as pointed out earlier, the interesting event occurs in the higher frequencies, especially in the final lobe; the two lobes are mirror images of each other. The spectra of Fig. 18 is the result of a 64-point FFT; if we apply higher number of FFT points (by padding more zeros), this mirror image event is still happening, only that it is more detailed, and every lobe is comprised of more number of points. An easily measurable discrimination factor would be the slope of the line connecting the peak and the valley of the last lobe at the highest frequency. In the case of Fig. 18, the discrimination factor is simply the subtraction of amplitude of ($N/2$)th point from the amplitude of ($N/2-1$)th point in an $N$-point FFT. The advantages of this approach are:

1. It is simple. Even with low number of points in FFT, this method works;
2. The amplitude of only two bins is enough for the discrimination. Therefore, there is no need for full-spectrum FFT calculation. Employing methods like Goertzel algorithm is enough to do the required measurements while keeping the process simple.

In Table 16, the FoMs for 12-bit resolution data are obtained using the method explained above, i.e., the slope of the line connecting the bins $N/2-1$ and $N/2$. A 64-point FFT is used for the data obtained using the DC440 digitizer with 12-bit resolution and at 420 MS/s, and a 32-bit FFT is used for the data obtained using the same digitizer but at 250 MS/s. As explained before, while this mirror image of the spectra in the higher frequency region is an easy approach to distinguishing neutrons and photons, this property cannot be used for the data obtained using digitizers featuring lower-resolution, e.g., 8 bits. For low-resolution data, the mirror image does not occur consistently with neutrons and photons. In high-resolution data, even the differences in low-frequency region can be easily used to discriminate the pulses, as Fig. 18 illustrates, but in low-resolution data, this difference is not enough for proper discrimination.

Another discriminating factor which could be exploited for any type of data, either the ones with low- or high-resolution, is the magnitude difference between the neutron and photon pulses; since the cut shown in Fig. 15 has higher average magnitude for neutrons than photons, this difference is also reflected in frequency domain (in zero frequency, i.e., the mean of the samples). In order to exploit this reflection, the second step in our method explained above, i.e., the subtraction of the mean of samples from every point, should be omitted. In our method, since we have used mean subtraction *after* windowing in step two, the zero frequency has zero value but the effect of windowing to decrease the spectral leakage is low, therefore, the zero frequency magnitude is leaked across the whole spectrum. In Sect.  6.3, we introduce a novel general method for better discrimination using the zero frequency.

**Table 16:** FoMs of the pulses obtained from various digitizers, applying FFT method

Digitizer	8-bit, 1 GS	8-bit, 2 GS	12-bit, 250 MS	12-bit, 420 MS
FoM	N/A	N/A	0.89	1.00

### Discrimination using variable window

In this Section, we will use a known principle to implement a variable window for discrimination purposes. The principle used here is introduced in [9]. Let $n(i)$ and $g(i)$ be two discrete-time functions, both normalized to unity, i.e.

4 $$\begin{aligned} \sum _in(i)=\sum _ig(i)=1 \end{aligned}$$

If we compute the time function of the relative difference between $n(i)$ and $g(i)$ (weights) as follows:

5 $$\begin{aligned} p(i)=\frac{g(i)-n(i)}{g(i)+n(i)} \end{aligned}$$

then an unknown function $u(i)$, close to either $n(i)$ or $g(i)$, can be identified as one of them by the sign of $S$ defined as:

6 $$\begin{aligned} S=\sum _ip(i)u(i) \end{aligned}$$

In this article, we use this principle to design a window for discrimination of neutrons and gamma-rays. In Eqs.  4, 5, and 6, if we replace $n(i)$ and $g(i)$ with neutron and gamma-ray pulses, respectively, then if $S<0$, the particle is identified as gamma-ray, and if $S>0$, as neutron.

According to Eq. 5, those parts of the neutron and photon signals that differ most will have greater weights and the similar parts will have negligible weights. The similar segments could have weights with large absolute values when they are very close to zero; but according to Eq. 6, the final effect is minimal. Since the leading edges and the end-tail segments of neutrons and gamma-rays have almost the same shape, there will be insignificant weights or effects for corresponding points when these segments are included. However this minimal improvement of the discrimination caused by these segments will help us better identify the particles in low energy region. Inclusion of these parts is directly related to the capabilities of the hardware at hand. Omitting these segments will have the benefit of fewer number of multiplications (based on Eq. 6), but a slight decrease in the quality of the results. For this work, the area of interest starts from the point where the leading edge hits the 1 % threshold level and the end point is a constant number of samples after this starting point for all signals, such that this interval covers a signal as much as possible.

In Eq. 5, a sample gamma-ray $g(i)$ and a sample neutron $n(i)$ are picked and used to build the weights. These samples need to be patterns representing the types of pulses contained in the whole data set. Therefore, more than one sample should be used for each pulse type to obtain better results. If we use $k$ number of pulses ($k>1$) from each radiation type to build the sample pulses required, then

7 $$\begin{aligned} g(i)&= \frac{\sum _{j=1}^kg_j(i)}{k}\nonumber \\ n(i)&= \frac{\sum _{j=1}^kn_j(i)}{k} \end{aligned}$$

**Fig. 19:**
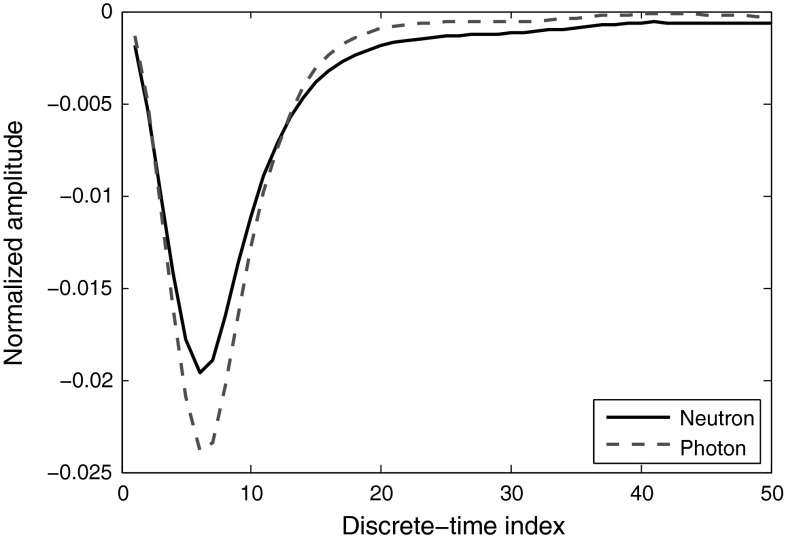
Segments of neutron and gamma-ray pulses, obtained from DC440 digitizer (12-bit resolution, 420 MS/s), when normalized to unity (using Eq. 4)

**Fig. 20:**
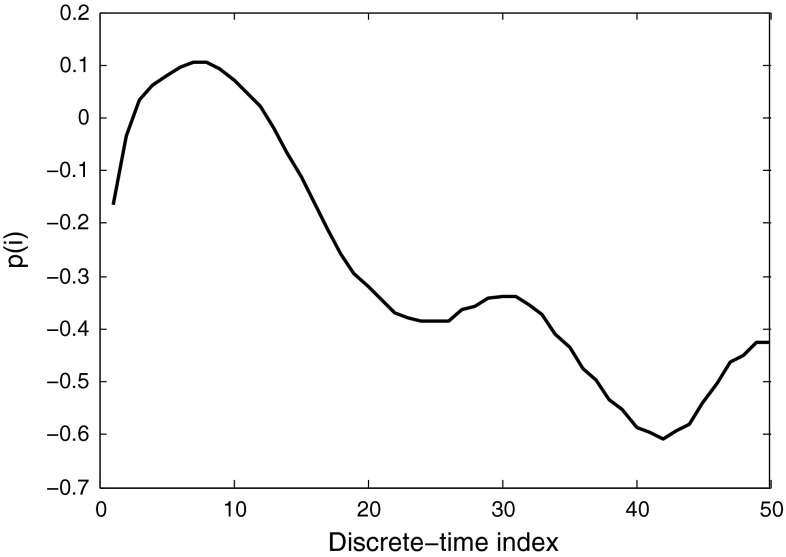
Weight function $p(i)$, obtained from Eq. 5 using the two signal segments shown in Fig. 19

Once every point of the two sample pulses are built using the Eq. 7, they are normalized to unity using the Eq. 4 (as Fig. 19 illustrates), and then applied to the Eq. 5 to build the weight sequence (as shown in Fig. 20).

We use the constant weight sequence $p(i)$, in conjunction with every arriving pulse, to scale a varying Hamming window. If $u(i)$ is the unknown pulse to be processed, it is passed along with $p(i)$ to Eq. 6 to compute $S$. As mentioned before, $S$ serves as the identifier for the pulse and hence can itself be used as counting/discriminating factor. However, $S$ could also be used to scale a window which is in turn used to count/discriminate. In order to preserve the scaling factor $S$ after applying window to the pulse, $S$ should be divided by $u(m)$, the median of the unknown sequence $u(i)$, where the peak of the window lies:

8 $$\begin{aligned} \text {Scale}=\frac{S}{u(m)} \end{aligned}$$

**Fig. 21:**
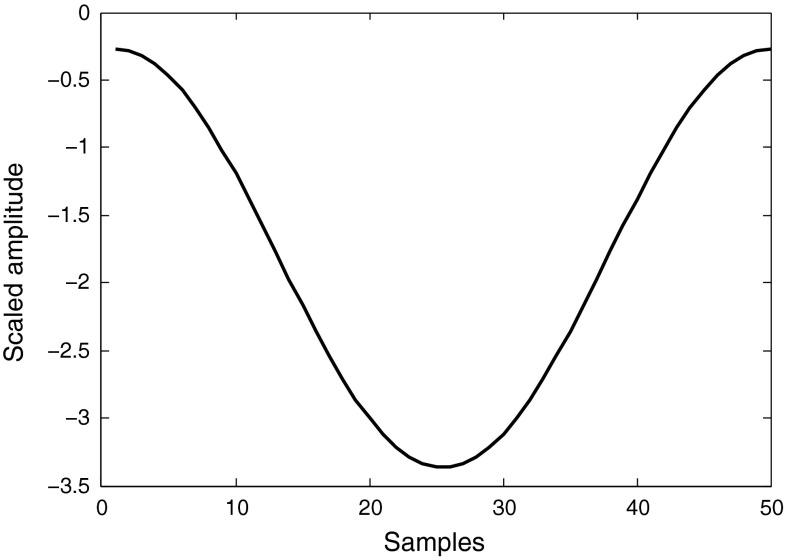
Hamming window with its amplitude scaled according to Eq. 8 for a specific pulse under process

However, Eq. 8 only scales the magnitude, not the sign, hence could be ignored. A sample Hamming window scaled in this manner for a specific neutron pulse is shown in Fig. 21.

**Fig. 22:**
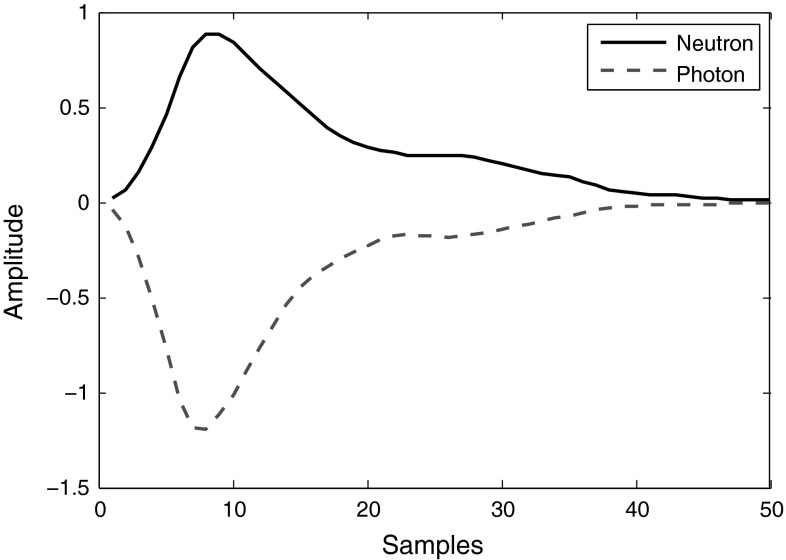
Segments of neutron and photon pulses after application of their corresponding scaled Hamming windows

A pulse is easily identified when its correspondingly-built window is applied to it. The direction of the pulse amplitude reveals its identity; Using Eq. 5, neutrons will have positive amplitudes while photons will have negative ones. This can be used to count the number of neutrons and photons in an experiment. Figure 22 shows two sample windowed neutron and photon pulses. Since the zero base-line is the separator between these signals, to find the efficiency of discrimination, an ideal factor to use would be the sum of a pulse sequence points. This sum is the DC value or the zero frequency of the windowed pulse. The choice of Hamming window for this application is clear now: pedestal raised property of this window pushes the two signal types far from each other on the two sides of zero baseline. However, the other window types like Hanning would perform well too.

**Fig. 23:**
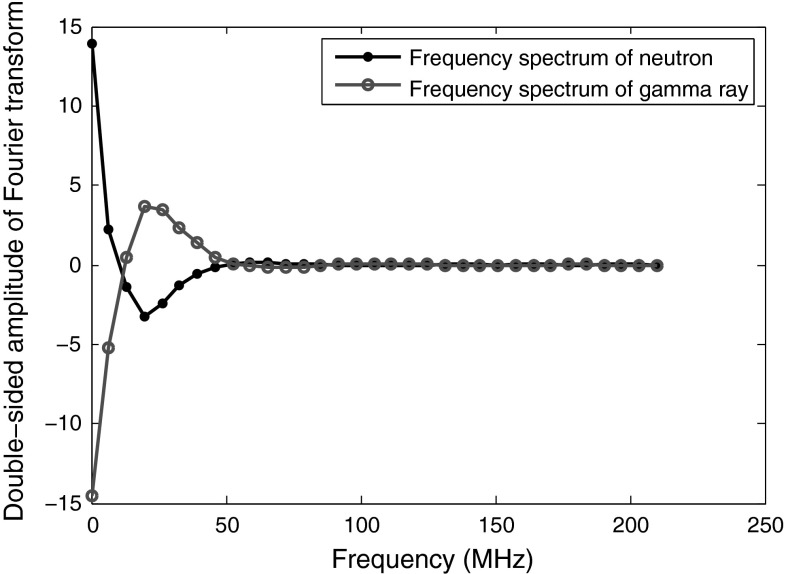
The double-sided amplitude spectra of the $\gamma$-ray and neutron events shown in Fig. 22, applying a 64-point FFT. The pulses are obtained using DC440 digitizer (12-bit resolution, 420 MS/s)

**Fig. 24:**
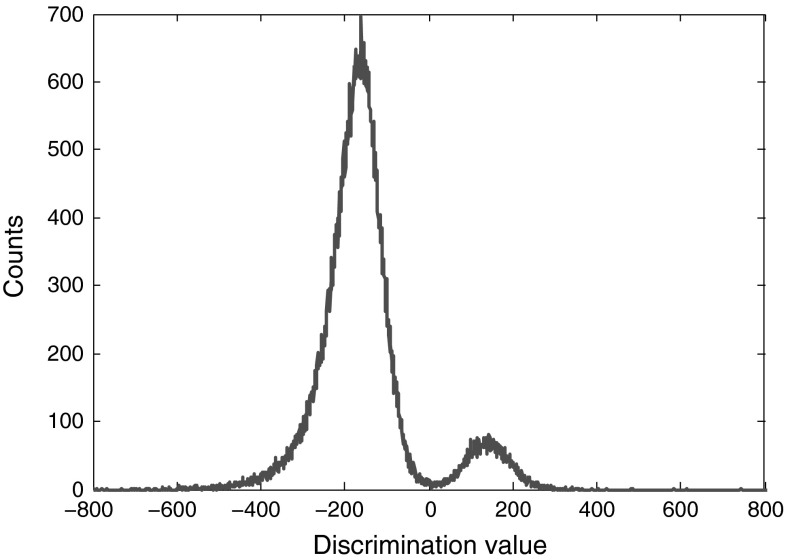
Discrimination of photon and neutron signals using variable window. The pulses were obtained using DC440 digitizer (12-bit resolution, 420 MS/s)

The double-sided amplitude spectra of neutron and gamma-ray signals in Fig. 22 are shown in Fig. 23; zero frequency can easily discriminate the two signal types. Figure 24 illustrates the experimental distribution plot of neutrons and photons for the data obtained from DC440 digitizer with 12-bit resolution and set at 420 MS/s frequency rate. As seen, the zero discrimination value is the separator here; neutrons have positive and gamma-rays have negative discrimination values. Table 17 shows the FoM (computed using Eq.  1) and neutron and photon counts for this data set. The discrimination quality is improved in this method compared to the application of FFT method, explained in Sect. 6.2. FoMs and pulse counts for the other data sets with different resolutions and frequency rates are shown in Table 18. While FFT method, explained previously, failed to discriminate low-resolution data, this method discriminates these pulses very efficiently.

**Table 17:** FoM and counts of the pulses obtained from DC440 digitizer

Data format	FoM	Neutron counts	Photon counts
12-bit, 420 MS/s	1.20	9149	90851

**Table 18:** FoMs and counts of the pulses obtained from DC440 and DP210 digitizers under different sampling rates

Data format	FoM	Neutron counts	Photon counts
12-bit, 250 MS/s	1.13	8807	91193
8-bit, 1 GS/s	1.12	9725	90275
8-bit, 2 GS/s	1.04	9204	90796

## Discussion

Two important factors affecting the FoM of a discrimination method are resolution and sampling rate of the digitizer. According to Nyquist criterion, the sampling rate must be greater than twice the bandwidth of continuous digitizer input signal. The FFT of the recorded neutron and photon signals indicates frequency components up to 100 MHz [10]. Therefore, the minimum necessary sampling frequency for neutron and photon signals is about 200 MS/s. The exact impact of the sampling rate on a specific separation method will depend on how the method functions. The separation method could mainly rely on the time difference, energy-level difference, or both time and energy-level differences of neutron and photon pulses, resulting in respectively high, low, and average impact of sampling rate on the separation quality. The estimation of exact effect of sampling rate on the FoM of a discrimination technique can be involved.

The factor with a greater impact on discrimination quality is digitizer resolution. The process of converting a discrete-time continuous-amplitude signal into a digital signal by expressing each sample value as a finite number of digits is called quantization. The resolution (or quantization step size) is the distance between two successive quantization levels. The error introduced in representing the continuous-valued signal by a finite set of discrete value levels is called quantization error or quantization noise. The quality of the digitizer output could be measured by signal-to-quantization noise ratio (SQNR). Since quantization errors of neutron and photon signals are almost uniformly distributed over the quantization interval, the following well-known Eq. [11] reliably estimates the quality of a $b$-bit digitizer output:

9 $$\begin{aligned} SQNR(dB)= 1.76+6.02b \end{aligned}$$

Equation 9 implies that SQNR increases approximately 6 dB for every bit added to the digitizer word length. This relationship gives the number of bits required by an application to assure a given signal-to-noise ratio.

The techniques presented in this article are all computationally simple; they exploit samples as early as possible in the life of the signals. This characteristic has several advantages. First, it helps alleviate pulse pile-up situation. This situation arises due to the random nature of the radiation, where a second event commonly occurs before the pulse from a previous event is completely in the output. This may cause false record of the second pulse’s energy levels. Since almost all the methods discussed in this article are fast, i.e., they try to detect the characteristics of either pulse type early in the lifetime of a pulse, there is less pulse pile-up problem when applying these methods. Second, typical embedded system technologies could be easily used for realization due to the simplicity of these methods. Third, in many industrial applications, neutron/gamma discrimination is required to be done in real-time fashion. Discrimination of the pulses through simple methods which exploit time-domain data (or quick algorithms in frequency domain) brings about quickness needed for real-time operations.

**Fig. 25:**
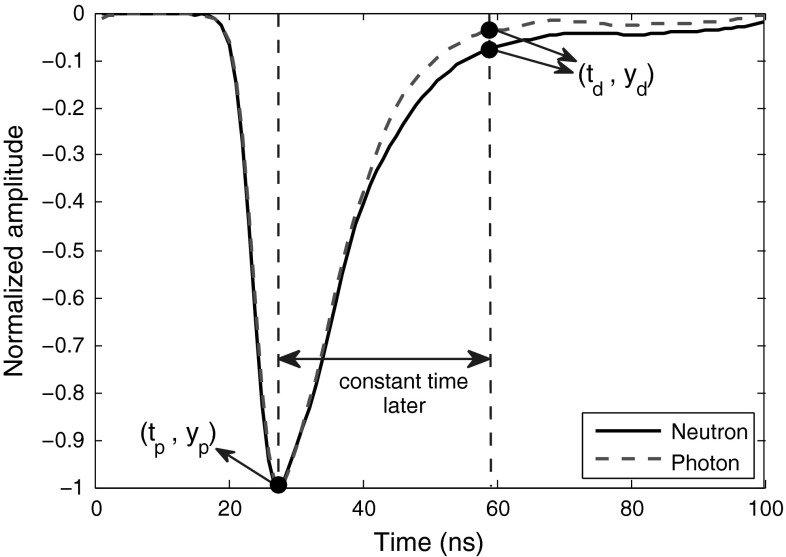
The points on smoothed neutron and photon signals used in “PGA” discrimination method

In Sect. 3.1, we applied classic rise-time method to the same pulses dataset as used for the other novel methods introduced in this paper. The FoM results shown in Tables 1, 2, 3 and 4 verify the performances of the novel methods in this article. As another verification, we apply the PGA method to the same pulses dataset. PGA method, introduced in [3], is recognized as an efficient $n/\gamma$ discrimination method with a high FoM. The slower decay of the light function of a scintillator for a neutron interaction than that for a $\gamma$-ray interaction is exploited in this method. The gradient between the peak amplitude and the amplitude a specified time after the peak amplitude (called the discrimination amplitude) on the trailing edge of the pulses are compared and used as the discrimination factor. Figure 25 illustrates the peak and discrimination amplitudes on neutron and photon signals. The gradient is calculated using

10 $$\begin{aligned} m=\frac{\Delta y}{\Delta t}=\frac{(y_p-y_d)}{(t_p-t_d)} \end{aligned}$$

where $m$, $y_p$, $y_d$, $t_p$, and $t_d$ are the gradient, the peak amplitude (which is a constant for normalized pulses), the discrimination amplitude, the time of peak amplitude occurrence, and the time of discrimination amplitude occurrence, respectively. For this work, we used some training pulses to locate the best discrimination amplitude, which occurred about $36\;ns$ after the peak of the pulse. In general, the optimal timing for the discrimination amplitude which makes the highest difference between the two radiation types is dependent on the scintillator properties and also on the PMT. The FoMs obtained are listed in Table 19. A comparison shows that almost all the novel methods introduced here are either better or at least have the same discrimination quality as the PGA method does.

**Table 19:** FoMs of “PGA” method for the pulses obtained from various digitizers

Digitizer	8-bit, 1 GS	8-bit, 2 GS	12-bit, 250 MS	12-bit, 420 MS
FoM	0.88	0.91	0.94	1.00

## Conclusion

In this article, we introduced several novel quick algorithms to discriminate the neutron and photon pulses captured in a mixed environment. These methods are appropriate for online measurements. Two digitizers, each featuring a different resolution and each set at two different sampling rates, were used to observe the reaction of each method to the data sampling conditions.

We categorized our discrimination techniques according to the type of measurement used to differentiate the neutron pulses from the photon ones. In general, in order to do the discrimination, the methods in each category could exploit the difference between neutrons and photons in their timing, or in amplitude, or both. In “Distance-Based Methods,” all these three cases were practiced separately. In “Area-Based Methods,” we only considered the experiment exploiting both differences in time and amplitude.

In “Angle-Based Methods,” we either exploited the difference in time, or energy, but not both. However, it is possible to make a combination of these methods, e.g., by addition of the angles generated by each method and use it as the discrimination factor, hence obtaining a better separation of the pulses. However, the FoMs of each method, either based on the horizontal difference or vertical, were efficient enough to stop short of more processing. The time-based method works for both low and high resolution data, and the energy-based method works for high resolution data.

Three other successful methods were also introduced. “Mean vs. Standard Deviation” method provides a high quality discrimination, almost irrespective of the resolution and sampling rate used to sample data. The “FFT” method, however, is promising only for the data recorded with high resolution. Finally, counting/discriminating using “Variable Window” always performs efficiently.

Depending on the features of the digitizer at hand, our recommendations for optimal discrimination methods, according to the results obtained in this article, are as follows:

- *Digitizer with high resolution (but not necessarily with high sampling rate)*: “Angle-based methods” (Sect. 5);
- *Digitizer with high sampling rate (but not necessarily with high resolution)*: “Distance on trailing edge method” (Sect. 3.6);
- *Digitizer with neither high resolution nor high sampling rate*: “Generalized rise-decay method” (Sect. 3.2), “Area-based method” (Sect. 4), and “Mean vs. std. method” (Sect. 6.1).
